# Digital workflow for fabrication of bespoke facemask in burn rehabilitation with smartphone 3D scanner and desktop 3D printing: clinical case study

**DOI:** 10.1186/s41205-022-00140-0

**Published:** 2022-05-04

**Authors:** Bushra Alhazmi, Feras Alshomer, Abdualziz Alazzam, Amany Shehabeldin, Obaid Almeshal, Deepak M. Kalaskar

**Affiliations:** 1 Division of Plastic Surgery, Department of Surgery, King Abdulaziz Medical City, Ministry of National Guard - Health Affairs (MNG-HA), King Abdullah International Medical Research Center (KAIMRC), Riyadh, Saudi Arabia; 2 Department of occupational therapy, King Abdulaziz Medical City, Ministry of National Guard Health Affairs, King Abdullah International Medical Research Center, Riyadh, Saudi Arabia; 3 UCL Institute of Musculoskeletal Sciences (IOMS), Division of Surgery and Interventional Science, Royal National Orthopaedic Hospital-NHS Trust, Stanmore, Middlesex, HA7 4LP UK

**Keywords:** 3D printing, 3D scanning, Orthosis, Burn, Scar, Face mask

## Abstract

We present a digital workflow for the production of custom facial orthosis used for burn scar management using smartphone three-dimensional (3D) scanner and desktop 3D printing. 3D facial scan of a 48-year-old lady with facial burn scars was obtained. 3D modeling with open-source programs were used to create facemask then 3D printed using rigid polylactic acid (PLA) filament and semi-rigid thermoplastic polyurethane (TPU). Conventional facemask was used as a control. Each mask was worn for 7 days. Primary outcomes were level of comfort, and adherence to treatment. The conventional facemask was the most convenient followed by the TPU-facemask (mean comfort score of 9/10 and 8.7/10, respectively). Patient’s compliance was high for both TPU and conventional masks, each was worn for at least 21 hours/day for 7 days. On the contrary, PLA-facemask was not well tolerated. The proposed digital workflow is simple, patient-friendly and can be adopted for resource-intensive healthcare.

**Supplementary Information:**

The online version contains supplementary material available at 10.1186/s41205-022-00140-0.

Burn injuries constitute a major health issue with nearly 11 million people worldwide requiring burn-related medical aid [1]. Facial burns have been estimated to constitute up to 50% of mild to moderate burns and were involved in around 50% of major burns. Facial burns can lead to debilitating injuries with functional, easthetic, and psychological sequelae [1]. One of the most common complications of facial burn is scar-related complications that includ hypertrophic scars (HS), contracture, and dyspigmentation. Facial appearance plays a vital role in one’s perception of identity and interaction with the society. Disfigurement following burn injury can affect one’s self-steam causing major psychological burden [2]. The initiation of a timely-introduced comprehensive rehabilitation program is a cornerstone in the management of facial burns to reduce possible complications and improve functional and aesthetic outcomes [2]. The rehabilitation process is typically thorough including but not limited to early and proper positioning of the head and neck following injury, performance of facial exercises, splinting where indicated, scar management, activity of daily living training, psycological support, family/care giver education, and social reintegration [2].

For scar management, pressure therapy and silicone products were the most commonly used modalities for prevention and treatment of HS [2]. Provision of an ideal pressure therapy is a challenging task giving the delicate nature and complex topography of the face. Pressure therapy is traditionally applied through the use of elastic pressure garments. Pressure garments, however, might fail to exert pressure over concave surfaces [2]. Moreover, areas near the holes created within the fabric around the eyes, nostrils, and mouth were subjected to less pressure [2]. When used alone, pressure garment have a low pressure which might deem the treatment ineffective [2]. An alternative tool to provide pressure therapy is with the use of customized pressure facemask which have shown to produce more evenly distributed pressure [2]. Traditionaly, the method of facemask creation included the use of alginate to take an impression of the patient’s face. The facemask is then molded based on the resulting plaster mold using a thermoplastic material. This usually requires a skilled therapist together with the needed time and logistics to facilitate such production [2]. We present a digital workflow using readily available smartphone based 3-dimentional (3D) scanning technology coupled with desktop 3D printing to produce bespoke facemask which can promote scar rehabilitation in facial burn survivors and pave the way to facilitate mask production.

## Material and methods

**Fig. 1:**
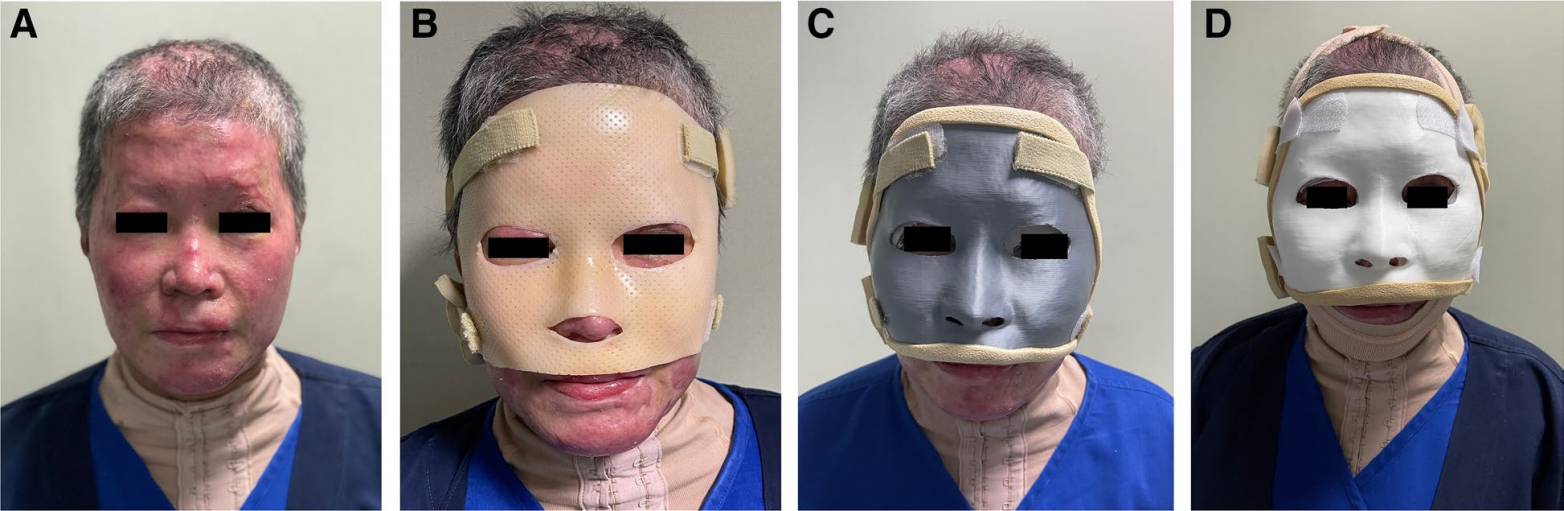
Shows frontal view of the patient’s profile with **A** prior to mask application. **B** While wearing the conventional mask. **C** While wearing the PLA rigid mask, and **D** while wearing the semi-rigid TPU mask. The patient gave written informed consent for their photos to be used for publication

This is a proof-of-concept study in which a digital workflow was implemented to produce a rigid and a semi-rigid 3D-printed facemask. These 3D-printed facemasks were compared with the traditional facemask typically used in burn rehabilitation. A 48-year-old female patient with a history of flame burn affecting 25% TBSA involving the face, neck, upper chest, back, and bilateral upper limb. For the facial burn injury, initial debridement and application of split thickness skin graft was done for the forehead and bilateral upper eyelid Fig. 1a. The face masks were applied during the initial rehabilitation period for scar management.

In our institution, traditional mask was fabricated by an experienced therapist using Orfilight® Atomic Blue NS (Orfit, Wijnegem, Belgium), a micro-perforated low-temperature thermoplastic splinting material. The thermoplastic sheet was activated by heating it at a temperature of 65–70 °C using a water bath. Once the material was activated, completely soft and can be hand handled after brief cooling period, it was then molded directly on the patient’s face to reach the desired shape. It was kept on the patient face till it fully cooled down and was sufficiently hardened. Padding of the edges was done using moleskin® and adjustable straps were affixed to the mask using self-adhesive material on the superior and inferior ends as shown in Fig. 1b.

For 3D printed masks, a threefold digital workflow was composed of 1) Acquisition of 3D data using smartphone-based 3D scanner to capture patient’s face, 2) 3D construction of personilized fasemask compatable with 3D printing using open-source CAD, and 3) printing the facemask using thermoplastic material on a desktop 3D printer.

### Acquisition of 3D data

We have utilized a smartphone (iPhone 12, Apple®) with facial recognition capabilities to perform the 3D scanning process of the patient’s face. The scanning was done utilizing Bellus3D FaceApp (Bellus3D® Campbell, CA) with the patient seated in upright position. Bellus3D FaceApp is a smartphone application that uses the phone facial recognition senser for 3D scanning and costs 0.99 US dollars per model for the export feature as a Alias Wavefront Object (.OBJ) file. Initially, few trials of scannning were done to familiarize the patient with the process. The scanning process acquired data in two axes (while patient turning the head to the sides and then by flexing and extending the neck) that helped acquiring more surface details of the patient’s face.

### Designing the facemask

The scanned model was exported in (.OBJ) format. The model was imported into Blender (Blender® Foundation, Vienna, Austria), an open-source CAD modeling software, in which sculpt mode was selected for further processing. “Mask extract” tool was then selected followed by manual highlighting of the area of interest which included the following zones: forhead, periorbital area, nose, and upper cutaneous lip as shown in Vid. 1, The resulting facemask was exported as. STL file. This partial facemask design was done to reduce the need for mask removal during meals, oral hygiene, or verbal communication. The facemask model was further edited using Meshmixer® (Autodesk Inc.), an open-source software, to give the STL file more volume and thickness to be printable. The file was then exported and was ready for 3D printing.

### 3D-printing and post-production manual refinements

The design was 3D printed using material extrusion on a desktop fused deposition modeling 3D printer (Ultimaker 2+, Ultimaker®, Geldermalsen, The Netherlands). Two materials were tested for mask fabrication. The rigid facemask was printed using a biodegradable polylactic acid (PLA) filament Fig. 1c. A semi-rigid mask was printed using a thermoplastic polyurethane (TPU) filament Fig. 1d.

Total time for mask fabrication and printing was recorded together with the cost per material. The print settings for the produced masks are summarized in Table 1.

**Table 1:** Summaries the printing settings for the produced masks. PLA; Polylactic acid, TPU; Thermoplastic Polyurethane, mm; millimeter, s; second

Mask type	TPU mask	PLA mask
**Printing parameters**	**• Printing parameters:** Same print parameter in the preloaded fine print profile in Cura 4.11.0 slicing software. Which are as follow: - Wall thickness 1.05 mm. - Top/bottom thickness 1.2 mm. - 10% infill density with grid pattern. - Print speed at 40 mm/s. - Everywhere support with overhang angle of 45 degrees.	**• Printing parameters:** Same print parameter in the preloaded fine print profile in Cura 4.11.0 slicing software. Which are as follow: - Wall thickness 1.05 mm. - Top/bottom thickness 0.8 mm. - 20% infill density with grid pattern. - Print speed at 50 mm/s. - Everywhere support with overhang angle of 50 degrees.

**Fig. 2:**
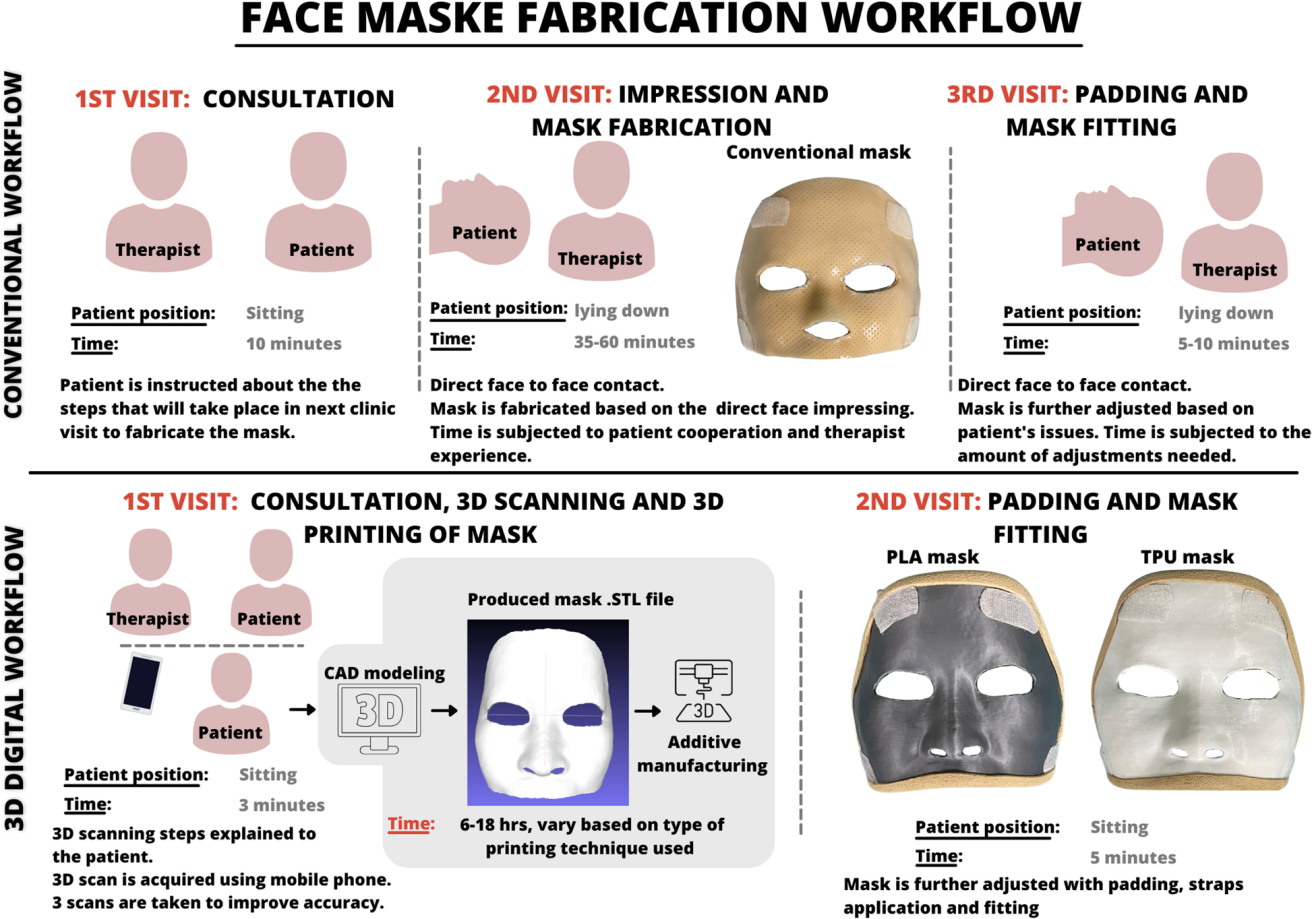
Summarizes the steps and clinical setup for conventional and 3D digital workflow involved in mask production. PLA; Polylactic acid, TPU; Thermoplastic Polyurethane

After the masks were printed, the resulting models were cleaned with the removal of its additional support material. Then padding of the edges of the masks was done using moleskin® to minimize skin friction. Two adjustable straps were affixed to each mask using self-adhesive material on the upper and lower ends. The steps of conventional and the digital workflow for mask production are summarized in Fig. 2.

All masks were worn with adjunct medical grade silicone sheet as a lining underneath. Each mask was scheduled to be worn for a period of 7 days. Total number of hours wearing the mask during the day and the presence of any side effects were recorded. A daily assessment of the patient’s level of comfort was done on a numerical scale of 1–10, with 10 being most comfortable. The patient was assessed daily and all data were collected and logged in excel sheet. The patient gave written informed consent for their photos and the supplementary video to be used for publication. The dataset generated during and/or analyzed during the current study are available from the corresponding author on reasonable requests.

### Statistical analysis

Data analysis was performed using Statistical Package for the Social Science (SPSS) in which analysis of variance (ANOVA) test was used to compare the continuous variables. A *p* value of < 0.05 was considered statistically significant.

## Results

At the end of 3 weeks period, the patient was interviewed and was asked about her experience starting from the fabrication process till the end of the assessment period.

### Digital workflow

For the mask fabrication starting from the initial interview, the face 3D scanning process took around 3 minutes including trial scans. The trial scans were done to familiarize the patient with the needed head motion for better quality scans. In the application that we have utilized, the scan file can be exported with the cost of 0.99 US dollars per model.

With regards to the fabrication process, we used the same 3D printer to print both the PLA and TPU masks. The total printing time was about 18 hours and 27 minutes for the semi-rigid TPU facemask, whereas for the rigid PLA facemask, the total printing time was about 15 hours and 55 minutes. For the conventional mask, the fabrication process was done in one clinic session, which took about 35 minutes.

**Fig. 3:**
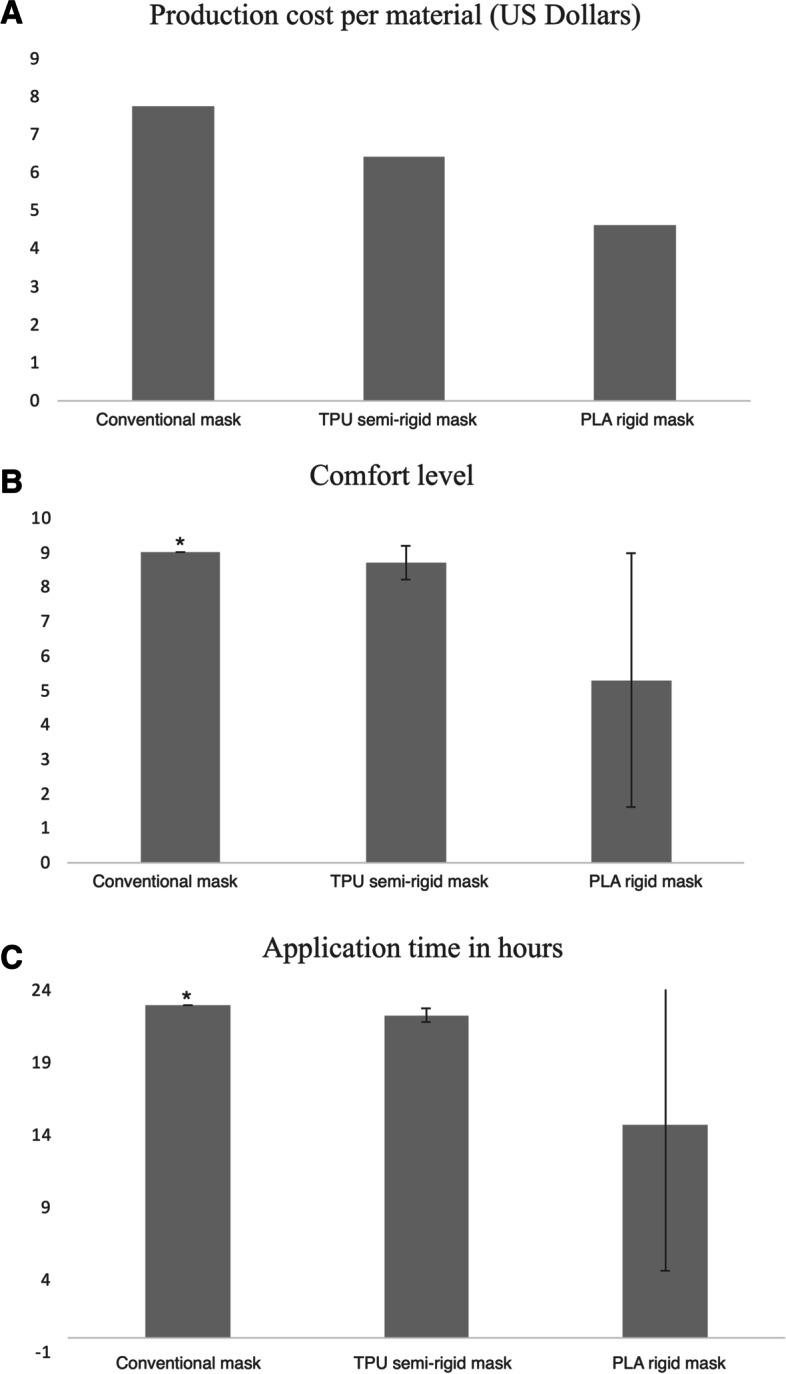
**a** Shows the production cost per material for each mask. Data is presented as US dollars. **b** Shows the overall out of 10 score comfort level of the patient while applying different masks. **c** Shows the application period of different face masks tested per day. Data is presented as means and standard deviation. *; *p* value of < 0.05

For the production cost, we looked at the cost per material. For the PLA rigid mask, the cost was 4.6 US dollars per material per mask. Whereas for the TPU semi-rigid mask the cost was 6.4 US dollars per material per mask. For the conventional mask, the cost was 7.73 US dollars per material per mask as shown in Fig. 3a. Data is summarized in Table 2.

**Table 2:** Summarizes the time needed to produce each mask together with cost of the used materials and the mask fabrication cost per material. PLA; Polylactic acid, TPU; Thermoplastic Polyurethane

Mask type	Conventional Mask	TPU mask	PLA mask
**Fabrication time**	• 35 minutes	• 18 hours and 27 minutes	• 15 hours and 55 minutes
**Raw material cost**	• Thermoplastic Orfilight Atomic Blue NS soft® microperforated 60 × 90 cm board costs about 90 US dollars.	• A 750 g spool of 2.8 mm TPU filament cost about 69.95 US dollars. • 68 g of material were used in the mask print.	• A 750 g spool of 2.8 mm PLA filament cost about 49.95 US dollars • 69 g of material were used in the mask print.
**Mask fabrication cost**	–	• 6.4 US dollars per material per mask	• 4.6 US dollars per material per mask

### Patients experience and comfort score

The patient was asked about her overall experience starting from mask fabrication and its usage for the trial period. The patient found that the use of facial 3D scanning was very comfortable as it took less time (about 3 minutes) without the need for direct physical contact with her skin. This was compared to the conventional method in which the patient mentioned certain difficulties associated with direct contact with her hypersensitive scars which was not the case with the scanning process.

When asked about the comfort level during mask application, the patient reported that the conventional mask was the most comfortable of all masks with mean comfort score of 9 out of 10. This was followed by semi-rigid TPU facemask with mean comfort score of 8.71 ± 0.48 out of 10. For the rigid PLA facemask, it was the least comfortable with mean score of 5.28 ± 3.68 out of 10 with statistically significant difference between the groups (*P* = .0075) Fig. 3b.

When asked about any issues with the tested masks during the application period, the patient mentioned that most of the issues were associated with the rigid PLA facemask (increased itchiness, sweating and discomfort), which led to discontinuation of its usage. (Questionnaire is attached as a supplementary material).

### Duration of mask usage

The patient adherence to the treatment by wearing the mask was recorded and analyzed. The patient was most adherent with the conventional facemask in which it was worn for a mean duration of 23 ± 0 hours per day for a total of 7 days with only breaks during meal and washing times. The semi-rigid TPU facemask was applied for a mean duration of 22.28 ± 0.48 hours per day for the total 7 days with similar breaks time as for the conventional mask. For the rigid PLA mask, it was only worn for 5 days as it was not tolerated well by the patient with mean application period of 14.7 ± 10.1 hours per day with statistically significant difference between all groups (*P* = 0.029) Fig. 3c.

## Discussion

3D printing has widespread use in medicine, and its accessibility and availability in medical practice has been further promoted through the advances in software development parallel with dramatic reduction in hardware cost [3–8]. While there are emerging applications in plastic surgery including the important role in full face transplantation, [4, 9] there is a paucity of evidence regarding personalized 3D printed orthosis pertaining for facial burn rehabilitation. Earlier reports described the application of 3D scanning and 3D printing to produce a replica of patient’s face with different clinical applications related to burn care [10, 11]. For which customized facemasks were manually fabricated based on the 3D printed face mold. For that, patient contact and associated anxiety and discomfort were eliminated.

More recently, the production of custom 3D printed facial orthosis was described. In a study by Wei et al., a portable 3D scanner was used to scan patients’ faces [12]. CAD-dervied facemask were then 3D printed using transparent rigid material, MED610 (Stratasys Ltd., Rehovot, Israel). For all recruited 10 patients, the mean scar thickness decreased significantly (*P* < 0.01) after 1 month of silicone-lined customized facemask application. The design of this mask was digitally modified to increase compression pressure over the hypertrophic scar, side of the cheeks, and middle of the chin area and decrease compression over the bony forehead. These subtle refinements (within 5 mm) were based on the analysis of a biomechanical model of the transparent facemask examining the pressure distribution over difference facial zones. Wei et al. also demonstrated the applicability of aforementioned approach in pediatric population [13]. In addition to HS prevention, Aguilar et al. described the use of personilized 3D-printed facemask for securing dermal substitute and skin graft [14]. Patient’s face was scanned using portable 3D scanner. The facemask was printed using a polylactic acid filament with a total cost of production of 100 US dollars. The facemask was reported to be well tolerated by the patient with no complications.

In this paper, we share our experience in utilizing smartphone-based 3D scanning for production of bespoke fasemask using CAD and 3D printing technology. In comparison to the published literature, one key advantage in this proposed digital framework is the utilization of smartphone-based 3D scanner. Several 3D data capturing systems exist on the market; however, the associated high costs (> 10,000$ typically) and lack of portability with some of the well-validated and commonly used systems might limit its widespread use [15].

With the recent advancements in smartphone cameras and sensors with improved spatial resolution and capture software, the feasibility to obtain a 3D scan were shown to have comparable precision to more well-established 3D capture systems. Rudy et al. investigated the precision of an iPhone X 3D scanner in facial analysis, they have reported a root mean square resolution value of 0.35 mm, which is better to other portable 3D scanners studied in the liturature [15]. Moreover, repeated scans of the same objects showed an average difference of < 0.5 mm providing reliable 3D data with relatively negligible cost [15]. We have implied the same concept with the additional implementation of open-source softwares for 3D data processing, together with mask design and construction to further increase the affordability. Furthermore, fused deposition modeling (FDM) 3D printing was used which is the most affordable and most commonly used consumer 3D printing technology [4].

Existing literature examined the use of 3D printed facial orthosis using rigid material [12–14]. However, in this pilot case study, in addition to rigid PLA-facemask, 3D-printed TPU-facemask was also assessed. The semi-rigid TPU-facemask was found to be more comfortable and better tolerated than PLA-facemask with nearly comparable results to the conventional modalities. Considerable attention should be given to patient’s comfort as it might affect compliance and treatment efficacy [11]. It is recommended that pressure therapy is applied between 18 and 24 hours a day [11]. Rebound hypertrophy can develop by virtue of patient non-compliance or premature treatment termination [11]. Larger clinical studies are needed to compare efficacy and convenience of rigid versus semi-rigid facemasks as well as 3D-printed facemasks.

We acknowledge the tradeoffs between costs, usability, comfort and time to print between our system and published data. Moreover, our technique may in theory be amenable for the pediatric population [16].

Establishing patient-friendly digital workflow can also prove useful in many situations for example in delivering care to patients in remote areas who do not have access to experienced occupational therapist or in situations of war with limited supplies and logistics in which patient’s facial scan can be obtained remotely. Additionally, this digital workflow does not represent a substitution to the current modalities but rather an aid in which it can also be used to prepare negative facial molds that traditional fabrications modalities can utilize for improved fitting of face masks without the need for direct physical contact with the patient.

Limitations of the current study include being a concept assessment study and its short duration. Although generally important, scar assessment tools were not applicable in this proof-of-concept in part due to short treatment periods allocated for each facemask, and the fact that the three facemasks were examined by the same patient rendered comparative efforts difficult. The difference in pressure exerted by three facemasks was not measured. Future efforts are directed to compare the efficacy of the TPU 3D-printed facemask in comparison to the traditional facemask in hypertrophic scar prevention and treatment.

## Conclusion

We present a digital workflow for production of custom facial orthosis used for facial burn scar management using smartphone 3D scanner and desktop-based 3D printing. This preliminary study describes a framework that is straightforward and uses off-the-shelf software, potentially overcoming numerous drawbacks associated with traditional manual approach. Clinical studies beyond the single patient reported are needed for further evaluation.

## Supplementary Information

**Additional file 1: Video 1**. Demonstrates the digital workflow and the CAD software process of making the 3D facemask model. The patient gave written informed consent for the supplementary video to be used for publication.

**Additional file 2: S1.** Patient questionnaire.

## Data Availability

Data related to the manuscript and further information are available upon request.

## References

[1] Clark C, Ledrick D, Moore A (2021). Facial Burns.

[2] Wei Y, Li-Tsang CWP (2017). Rehabilitation of Patients with Facial Burn Injury: Principles and Practice Experiences. JSM Burns Trauma.

[3] Calvo-Haro JA, Pascau J, Asencio-Pascual JM (2021). Point-of-care manufacturing: a single university hospital’s initial experience. 3D Print Med.

[4] Chae M, Rozen W, McMenamin P, Findlay M, Spychal R, Hunter-Smith D. Emerging applications of bedside 3D printing in plastic surgery. Front Surg. 2015;2:25. 10.3389/fsurg.2015.00025 PMC4468745 26137465

[5] Meglioli M, Naveau A, Macaluso GM (2020). 3D printed bone models in oral and cranio-maxillofacial surgery: a systematic review. 3D Print Med.

[6] Galstyan A, Bunker MJ, Lobo F (2021). Applications of 3D printing in breast cancer management. 3D Print Med.

[7] De La Peña A, De La Peña-Brambila J, Pérez-De La Torre J (2018). Low-cost customized cranioplasty using a 3D digital printing model: a case report. 3D Print Med.

[8] Kadakia RJ, Wixted CM, Allen NB (2020). Clinical applications of custom 3D printed implants in complex lower extremity reconstruction. 3D Print Med.

[9] Mitsouras D, Liacouras P, Imanzadeh A, Giannopoulos AA, Cai T, Kumamaru KK, George E, Wake N, Caterson EJ, Pomahac B, Ho VB, Grant GT, Rybicki FJ (2015). Medical 3D Printing for the Radiologist. Radiographics..

[10] Lin J, Nagler W (2003). Use of surface scanning for creation of transparent facial orthoses. Burns..

[11] Pilley M, Hitchens C, Rose G, Alexander S, Wimpenny D (2011). The use of non-contact structured light scanning in burns pressure splint construction. Burns..

[12] Wei Y, Wang Y, Zhang M, Yan G, Wu S, Liu W (2018). The application of 3D-printed transparent facemask for facial scar management and its biomechanical rationale. Burns..

[13] Wei Y, Li-Tsang C, Liu J, Xie L, Yue S (2017). 3D-printed transparent facemasks in the treatment of facial hypertrophic scars of young children with burns. Burns..

[14] Aguilar H, Mayer H (2019). A New Method for Securing Dermal Substitutes and Skin Grafts to Difficult Portions of the Face Using a Custom 3D-Printed Facemask. J Burn Care Res.

[15] Rudy H, Wake N, Yee J, Garfein E, Tepper O (2020). Three-Dimensional Facial Scanning at the Fingertips of Patients and Surgeons: Accuracy and Precision Testing of iPhone X Three-Dimensional Scanner. Plastic Reconstruct Surg.

[16] Alazzam A, Aljarba S, Alshomer F, Alawirdhi B (2021). The Utility of Smartphone 3D Scanning, Open-Sourced Computer-aided Design, and Desktop 3D Printing in the Surgical Planning of Microtia Reconstruction: a Step by Step Guide and Concept Assessment. JPRAS Open.

