# Supplementary material for: Digital workflow for fabrication of bespoke facemask in burn rehabilitation with smartphone 3D scanner and desktop 3D printing: clinical case study
Source: 3D Print Med. 2022 May 4;8:12. doi: 10.1186/s41205-022-00140-0 (PMC9069819; doi:10.1186/s41205-022-00140-0)
Supplement: Supplementary file 2 — Additional file 2: S1. Patient questionnaire. [file 41205_2022_140_MOESM2_ESM.docx]

| Face 3D scan | |
| --- | --- |
| How comfortable was the 3D scanning experience from 1-10?  (1-least,10-most) |  |

Please answer the following questions on a daily basis

| White Mask | | | |
| --- | --- | --- | --- |
| Days | How comfortable was the mask from 1-10 (1-least,10-most)? | How many hours were you wearing the mask? Reason for removal? | Any associated side effects noted? specify if any |
| Day 1  Date: |  |  |  |
| Day 2 |  |  |  |
| Day 3 |  |  |  |
| Day 4 |  |  |  |
| Day 5 |  |  |  |
| Day 6 |  |  |  |
| Day 7 |  |  |  |

| Grey Mask | | | |
| --- | --- | --- | --- |
| Days | How comfortable was the mask from 1-10 (1-least,10-most)? | How many hours were you wearing the mask? Reason for removal? | Any associated side effects noted? specify if any |
| Day 1  Date: |  |  |  |
| Day 2 |  |  |  |
| Day 3 |  |  |  |
| Day 4 |  |  |  |
| Day 5 |  |  |  |
| Day 6 |  |  |  |
| Day 7 |  |  |  |

| Conventional Mask | |
| --- | --- |
| How comfortable was the mask fabrication experience from 1-10 (1-least,10-most)? |  |

Please answer the following questions on a daily basis

| Conventional Mask | | | |
| --- | --- | --- | --- |
| How comfortable was the mask fabrication experience from 1-10 (1-least,10-most)? | | |  |
| Days | How comfortable was the mask from 1-10 (1-least,10-most)? | How many hours were you wearing the mask? Reason for removal? | Any associated side effects noted? specify if any |
| Day 1  Date: |  |  |  |
| Day 2 |  |  |  |
| Day 3 |  |  |  |
| Day 4 |  |  |  |
| Day 5 |  |  |  |
| Day 6 |  |  |  |
| Day 7 |  |  |  |
